# Experimental infection of mice with noncytopathic bovine viral diarrhea virus 2 increases the number of megakaryocytes in bone marrow

**DOI:** 10.1186/s12985-018-1030-7

**Published:** 2018-07-28

**Authors:** Kyung-Hyun Lee, Du-Gyeong Han, Suhee Kim, Eun-Jin Choi, Kyoung-Seong Choi

**Affiliations:** 1 Animal Disease Diagnostic Division, Animal and Plant Quarantine Agency, Gimcheon, 39660 Republic of Korea; 2 Department of Animal Science and Biotechnology, College of Ecology and Environmental Science, Kyungpook National University, Sangju, 37224 Republic of Korea; 3 Animal Disease & Biosecurity Team, National Institute of Animal Science, Rural Development Administration, Wanju-Gun, 55365 Republic of Korea

**Keywords:** Bovine viral diarrhea virus, Immunohistochemistry, Thrombocytopenia, Megakaryocyte

## Abstract

**Background:**

Bovine viral diarrhea virus (BVDV) causes significant economic losses worldwide in the cattle industry through decrease in productive performance and immunosuppression of animals in herds. Recent studies conducted by our group showed that mice can be infected with BVDV-1 by the oral route. The purpose of this study was to assess the clinical signs, hematological changes, histopathological lesions in lymphoid tissues, and the distribution of the viral antigen after oral inoculation with a Korean noncytopathic (ncp) BVDV-2 field isolate in mice.

**Methods:**

Mice were orally administered a low or high dose of BVDV-2; blood and tissue samples were collected on days 2, 5, and 9 postinfection (pi). We monitored clinical signs, hematological changes, histopathological lesions, and tissue distribution of a viral antigen by reverse transcription-polymerase chain reaction (RT-PCR) and immunohistochemistry (IHC) and then compared these parameters with those in ncp BVDV-1 infections.

**Results:**

None of the infected mice developed any clinical signs of the illness. Significant thrombocytopenia was found in both low- and high-dose-inoculated mice on day 2 pi. Leukopenia was apparent only in low-dose-inoculated mice on day 2 pi, whereas lymphopenia was not observed in any ncp BVDV-2-infected animal. Viral RNA was found in the spleen in of low- and high-dose-inoculated mice by RT-PCR. According to the results of IHC, the viral antigen was consistently detected in lymphocytes of bone marrow and spleen and less frequently in bronchus-associated lymphoid tissue (BALT), mesenteric lymph nodes, and Peyer’s patches. Despite the antigen detection in BALT and mesenteric lymph nodes, histopathological lesions were not observed in these tissues. Lympholysis, infiltration by inflammatory cells, and increased numbers of megakaryocytes were seen in Peyer’s patches, spleens, and bone marrow, respectively. In contrast to ncp BVDV-1 infection, lympholysis was found in the spleen of ncp BVDV-2-infected mice. These histopathological lesions were more severe in high-dose-inoculated mice than in low-dose-inoculated mice.

**Conclusions:**

Our results provide insight into the pathogenesis of ncp BVDV-2 infection in mice. Collectively, these results highlight significant differences in pathogenesis between ncp BVDV-1 and ncp BVDV-2 infections in a murine model.

## Background

Bovine viral diarrhea virus (BVDV) is an important viral pathogen of cattle and causes significant economic losses worldwide. BVDV, a member of the *Pestivirus* genus in the family *Flaviviridae*, is divided into two species, BVDV-1 and BVDV-2, on the basis of antigenic and genetic differences [1]. Both species consist of two biotypes, cytopathic (cp) and noncytopathic (ncp), on the basis of their ability to cause changes in the appearance of the cytoplasm of infected cells. BVDV is known to acutely infect immunocompetent animals, results in a mild subclinical to severe, even fatal, systemic clinical disease that can lead to reproductive problems such as embryonic death, abortions, and still births if pregnant cows are infected [2–4]. The most important source for direct and indirect transmission of BVDV is driven by persistently infected (PI) animals that were infected in utero*,* and these PI animals shed virus continuously throughout their lifespan.

BVDV can infect a wide range of hosts, including cattle, sheep, swine, goats, and wild ungulates [5–7]. Antibodies against BVDV have been detected in wild and domesticated ruminants and porcine species [8–11]. Recent studies conducted by our group showed that mice can be infected with BVDV-1 via the oral route; however, shedding of the virus by mice was not detected [12, 13]. In addition, hematological changes, histopathology, and viral antigen distributions were quite different between cp BVDV-1- and ncp BVDV-1-inoculated mice [12, 13].

In cattle, ncp BVDV-2 infection may cause more severe clinical signs, as well as decreases in the numbers of leukocytes, lymphocytes, and thrombocytes as compared to ncp BVDV-1 infection [14–17]. The outcomes of BVDV infection can differ depending on the BVDV species used. Little information is currently available regarding the association between pathology and specific genotypes of BVDV in a murine model. Our previous study evaluated platelet counts, histopathological lesions, and the presence of a viral antigen in mice during acute infection with ncp BVDV-1, and these results were similar to those observed in cattle. Therefore, the objective of this study was to assess the clinical signs, hematological changes, histopathological lesions in lymphoid tissues, and the distribution of the viral antigen after oral inoculation with a Korean ncp BVDV-2 field isolate in mice, and to compare these results with the virus distribution and histopathological findings in ncp BVDV-1 infections.

## Methods

### BVDV culture and mouse infections

Specific pathogen-free BALB/c mice (6−8 weeks old) were purchased from Central Laboratory Animal, Inc. (Seoul, Korea). All the animals were maintained under pathogen-free conditions and handled in accordance with the guidelines and protocols approved for these experiments by the Kyungpook National University Institutional Animal Care and Use Committee.

The ncp BVDV-2 (BVDV-2a, 11F001) strain and Madin-Darby bovine kidney (MDBK) cells used in this study were provided by the Animal and Plant Quarantine Agency in the Republic of Korea [18]. On receipt, MDBK cells were tested by PCR for mycoplasma contamination (Takara Bio Inc., Japan) and were found to be free of mycoplasma. This virus strain was obtained after 5 days of culture, one freeze-thaw cycle, and centrifugation at 1900 × *g* for 10 min to remove large cellular debris. The supernatant was then frozen at −80°C in aliquots until inoculation into mice. The virus was titrated in MDBK cell cultures, and the 50% tissue culture infective dose (TCID_50_) was calculated.

Twenty-four mice were assigned to the ncp BVDV-2 infection group, and six mice were used in the mock infection group; 30 mice were subjected to each experiment. Mice (*n* = 24) were orally challenged with either a low dose (*n* = 12; 4 × 10^5^ TCID_50_) or a high dose (n = 12; 1.2 × 10^6^ TCID_50_) of BVDV-2. Mock-infected mice were orally administered 0.4 mL of a tissue culture medium (Minimum Essential Medium; Life Technologies Corp., Carlsbad, CA, USA). Experiments were repeated three times to confirm reproducibility. A total of 90 mice were orally inoculated.

### Hematological examination, reverse transcription-polymerase chain reaction (RT-PCR), and histopathological analysis

During the experiments, the animals were monitored daily for behavioral characteristics, such as appetite, ruffled fur, and reduced activity. On days 2, 5, and 9 postinfection (pi), two mock-infected and eight ncp BVDV-2-infected mice were weighed and euthanized with CO_2_ gas to collect blood and tissue samples. Vacutainer tubes containing EDTA (Becton Dickinson, Franklin Lakes, NJ, USA) were employed for blood collection to prevent coagulation. Total and differentiated leukocytes (basophils, eosinophils, neutrophils, lymphocytes, and monocytes) and thrombocytes were counted on a VetScan HM5 Hematology System (Abaxis, Union, CA, USA).

The spleen, lungs, liver, kidneys, heart, intestines, mesenteric lymph nodes, Peyer’s patches, and femurs were removed at necropsy. Some of these organs and tissues were used for analysis of BVDV by RT-PCR. Total RNA was extracted from tissues with the RNeasy Mini Kit (Qiagen, Hilden, Germany). RT-PCR was performed as previously described [19]. Amplification of 5′-untranslated region was carried out with primers 324 and 326: 324 [F], 5′-ATG CCC WTA GTA GGA CTA GCA-3′ (W = A or T) and 326 [R], 5′-TCA ACT CCA TGT GCC ATG TAC-3′. The predicted size of the amplicon was 288 bp. The mouse inoculum (ncp BVDV-2a) and distilled water were served as a positive and negative control, respectively.

The remaining tissues were fixed in 10% buffered formalin, processed, embedded in paraffin, and prepared as 5-μm sections; one section of each tissue was stained with hematoxylin and eosin (H&E). Histopathological changes were investigated. Megakaryocytes were counted, and the result was expressed as the mean from five sections per slide at 200-fold magnification. All evaluations were conducted independently by two pathologists.

### Immunohistochemistry (IHC)

For BVDV antigen detection, 5-μm-thick paraffin-embedded tissue sections were deparaffinized and hydrated through a graded alcohol series before heat-induced antigen retrieval in 10 mM sodium citrate buffer (pH 6) for 30 min. A primary anti-BVDV monoclonal antibody (DMAB28412; Creative Diagnostics, Shirley, NY, USA) was used according to the manufacturer’s instructions. Next, the tissue sections were stained with a biotinylated anti-mouse IgG antibody (Vector Laboratories, Inc., Burlingame, CA, USA) for 1 h at room temperature, washed, and incubated with the VECTASTAIN ABC Reagent (Vector Laboratories) for 30 min. After washing, the tissue sections were allowed to react with a peroxidase substrate solution (Vector), rinsed, counterstained, mounted, examined by light microscopy, and photographed. Negative control slides were prepared by staining with isotype-matched IgG at the same dilution as that used for the primary antibody.

### Statistical analysis

Data are expressed as the mean ± standard error of the mean. Each value was the result of three independent experiments. Statistical analyses were performed in GraphPad Prism 5.0 (GraphPad Software Inc., San Diego, CA, USA). Statistically significant differences between low- or high-dose-infected and mock-infected mice at each time point (days 2, 5, and 9) were determined by one-way analysis of variance (ANOVA) followed by Dunnett’s post hoc test for multiple comparisons. Data with *P* values < 0.05 were considered significant.

## Results

### Clinical signs

**Fig. 1:**
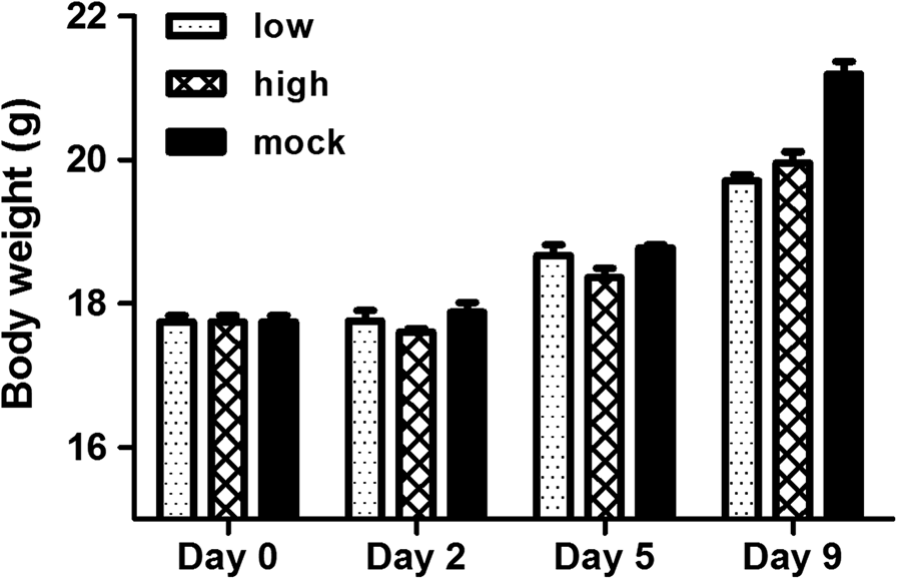
Body weight of mice infected with a low (4 × 10^5^ TCID_50_) or high dose (1.2 × 10^6^ TCID_50_) of ncp BVDV-2 or mice with the mock infection. Data are presented as the mean ± SEM of three independent experiments. Statistical analyses were performed by one-way analysis of variance in GraphPad Prism 5.0 software. A difference with a *P* value less than 0.05 compared to the mock-infected group was considered significant

No clinical signs, such as ruffled fur, reduced activity, reluctance to move, or crouching, and no weight loss were observed in ncp BVDV-2-infected mice. Body weights of high-dose-inoculated mice slightly decreased on days 2 and 5 pi as compared to low-dose-inoculated animals. Weight gain in high-dose-inoculated mice was seen on day 9 pi. Contrary to the high-dose-inoculated mice, body weights of the low-dose-inoculated mice did not diminish and were similar to those of mock-infected animals until day 5 pi and then decreased on day 9 pi as compared to mock infection. Body weights steadily increased among both low- and high-dose-inoculated mice during the experiment (Fig. 1).

### Hematological analysis

**Fig. 2:**
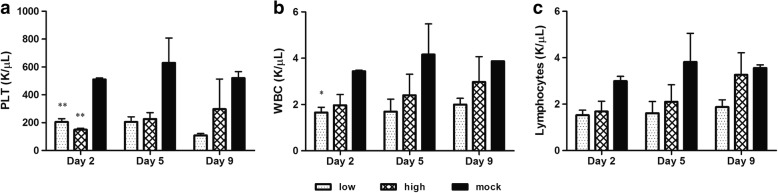
Mean numbers of platelets (**a**), white blood cells (**b**), and lymphocytes (**c**) in mice infected with a low (4 × 10^5^ TCID_50_) or high dose (1.2 × 10^6^ TCID_50_) of ncp BVDV-2 or in mice with the mock infection. Data are presented as the mean ± SEM of three independent experiments. Statistical analyses were performed by one-way analysis of variance in GraphPad Prism 5.0 software; * *P* < 0.05, ** *P* < 0.01, and *** *P* < 0.001 as compared with the mock-infected group

Platelet counts decreased in all BVDV-inoculated mice compared to those in the mock-infected animals. In low-dose-inoculated mice, the number of platelet decreased on day 2 pi (*P* < 0.01), remained stable until day 5 pi, and then decreased again on day 9 pi, whereas platelet counts in the high-dose-inoculated mice were the lowest on day 2 pi (*P* < 0.01) and then gradually increased (Fig. 2a). In both low- and high-dose-inoculated mice, significant thrombocytopenia was observed only on day 2 pi. The decrease in platelet counts was more prominent in low-dose- than in high-dose-inoculated mice (Fig. 2a). The number of circulating leukocytes in both low- and high-dose-inoculated mice gradually increased until day 9 pi. The numbers of leukocytes markedly decreased in low-dose-inoculated mice on day 2 pi (*P* < 0.05, Fig. 2b), compared to those in mock-infected mice. The number of lymphocytes was generally lower in low-dose-inoculated mice than in high-dose-inoculated mice (Fig. 2c). Lymphocyte counts in high-dose-inoculated mice increased consistently until day 9 pi. Although lymphocyte counts were lower in low-dose-inoculated mice than in high- dose-inoculated animals, this difference was not statistically significant.

### Detection of BVDV

**Fig. 3:**
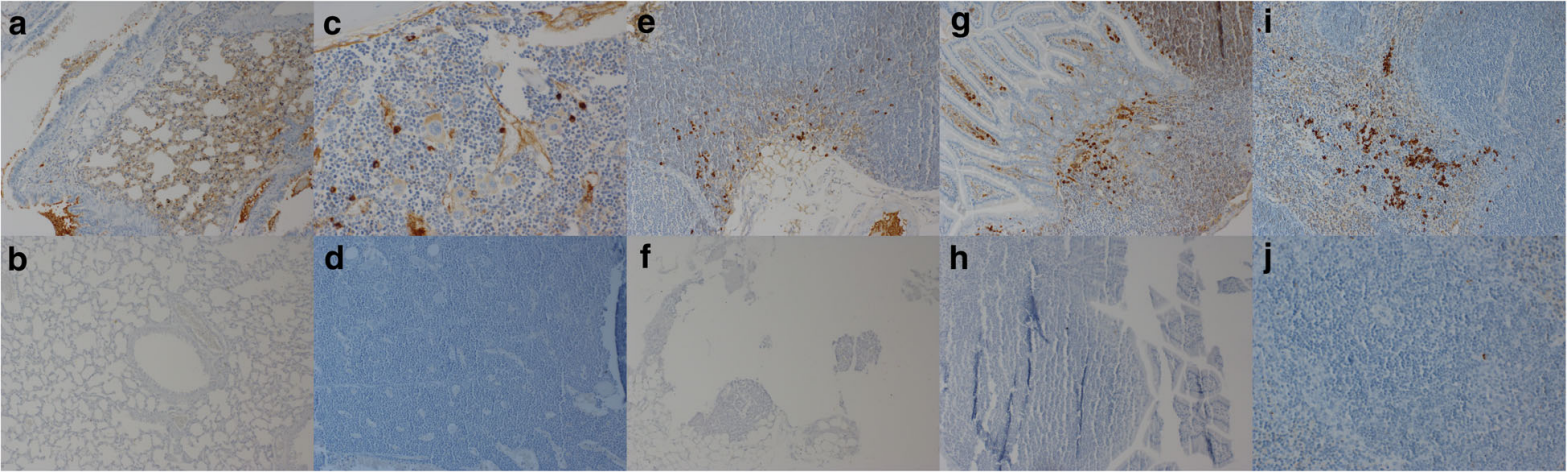
IHC of the viral antigen on day 5 pi in mice inoculated orally with a low (4 × 10^5^ TCID_50_) of ncp BVDV-2 or in mice with the mock infection. The viral antigen was detected in lymphocytes of bronchus-associated lymphoid tissue (BALT) (**a**), bone marrow (**c**), mesenteric lymph nodes (**e**), Peyer’s patches (**g**), and spleen (**i**) (black arrows) (original magnification: 200×). The viral antigen was not detected in the samples of BALT (**b**), bone marrow (**d**), mesenteric lymph nodes (**f**), Peyer’s patches (**h**), and spleen (**j**) samples of mock-infected mice. Magnification in all images: ×200. All the experiments were repeated three times

The presence of viral RNA in tissue samples was determined by RT-PCR. Viral RNA was detected only in the spleen (Table 1). Among low-dose-inoculated mice, viral RNA was found in only two animals on day 2 pi and then was detected in all the animals over time, whereas among high-dose-inoculated mice, viral RNA was not found in all animals during the experiment (Table 1). The distribution of the viral antigen was also evaluated by IHC in each BVDV-inoculated mouse (Table 2). In mock-infected mice, the viral antigen was not detected in any tissue samples. The viral antigen was detected in lymphocytes of bronchus-associated lymphoid tissue (BALT; Fig. 3a), bone marrow (Fig. 3c), mesenteric lymph nodes (Fig. 3e), Peyer’s patches (Fig. 3g), and spleen (Fig. 3i). As presented in Table 2, the viral antigen was consistently detectable in bone marrow and spleen of all BVDV-infected mice during the experiment, whereas the viral antigen was not found in BALT, mesenteric lymph nodes, and Peyer’s patches on day 9 pi. In BALT, the viral antigen was detected only in one high-dose-inoculated mouse on day 5 pi. In mesenteric lymph nodes, the viral antigen was found in two low-dose-inoculated mice and one high-dose-inoculated mouse on days 2 and 5 pi, respectively. In Peyer’s patches, the viral antigen was detected only in one low-dose-inoculated mouse on day 2 pi; the viral antigen in this tissue was detected in one high-dose-inoculated mouse on days 2 and 5 pi, respectively, and then disappeared on day 9 pi.

**Table 1:** BVDV detection results (RT-PCR) in the spleen from mice inoculated orally with a low or high dose of ncp BVDV-2

	L1	L2	L3	L4	H1	H2	H3	H4
Day 2	+	+	−	−	+	−	+	+
Day 5	+	+	+	+	+	+	+	−
Day 9	+	+	+	+	+	−	+	+

Eight mice were euthanized at each time point. Four mice were inoculated with a low dose, and four were inoculated with a high dose of the virus. Three independent experiments were conducted, and the results were the same. The data are representative of three independent experiments

L: low dose (4 × 10^5^ TCID_50_); H: high lose (1.2 × 10^6^ TCID_50_)

“−”: not detected, “+”: detected

**Table 2:** BVDV detection by IHC in mice inoculated orally with a low or high dose of ncp BVDV-2

Day	Day 2								Day 5								Day 9							
	L1	L2	L3	L4	H1	H2	H3	H4	L1	L2	L3	L4	H1	H2	H3	H4	L1	L2	L3	L4	H1	H2	H3	H4
Tissues																								
BALT	−	−	−	−	−	−	−	−	−	−	−	−	−	−	−	+	−	−	−	−	−	−	−	−
Bone marrow	+	+	+	+	+	+	+	+	+	+	+	+	+	+	+	+	+	+	+	+	+	+	+	+
Mesenteric lymph node	−	−	+	+	+	−	−	−	−	−	+	+	−	−	−	+	−	−	−	−	−	−	−	−
Peyer’s patches	−	−	−	+	+	−	−	−	−	−	−	−	−	−	−	+	−	−	−	−	−	−	−	−
Spleen	+	+	+	+	+	+	+	+	+	+	+	+	+	+	+	+	+	+	+	+	+	+	+	+

Eight mice were euthanized at each time point. Four mice were inoculated with a low dose, and four were inoculated with a high dose of the virus. Three independent experiments were conducted and the results were the same. The data are representative of three independent experiments

L: low dose (4 × 10^5^ TCID_50_); H: high lose (1.2 × 10^6^ TCID_50_)

“−”: not detected, “+”: detected

BALT: bronchus-associated lymphoid tissue

### Histopathological findings

**Fig. 4:**
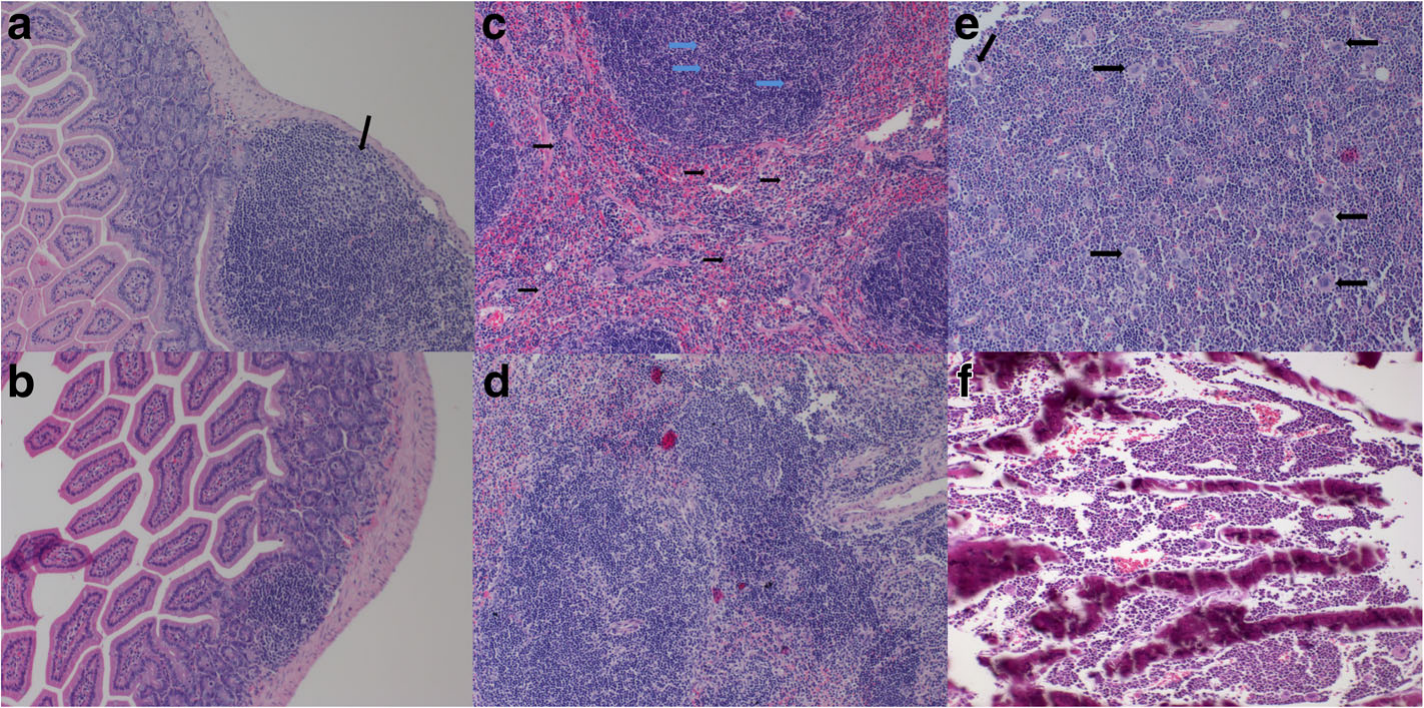
Histopathological features of Peyer’s patches, spleen, and bone marrow on day 5 pi in mice inoculated orally with a low (4 × 10^5^ TCID_50_) of ncp BVDV-2 or in mice with the mock infection. Lympholysis was observed in Peyer’s patches (**a**) and spleen (**c**, blue arrow). Monocytes infiltrated the red pulp, and the number of megakaryocytes increased in the spleen (**c**, black arrow), and in bone marrow (**e**), respectively. No histological changes were observed in Peyer’s patches (**b**), spleen (**d**), and bone marrow (**f**) of mock-infected mice. Magnification in all images: ×200. All the experiments were repeated three times

**Fig. 5:**
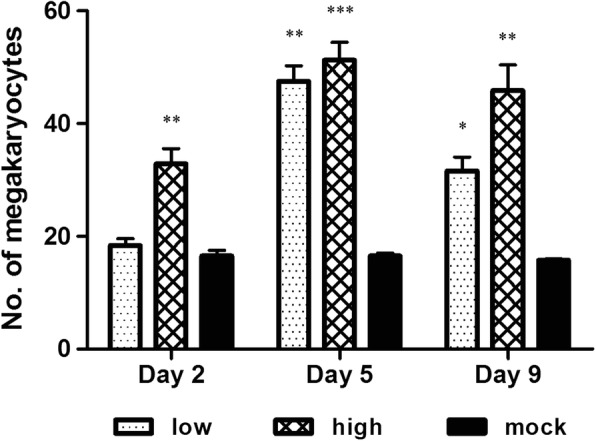
The numbers of megakaryocytes in bone marrow of mice inoculated orally with a low (4 × 10^5^ TCID_50_) or high dose (1.2 × 10^6^ TCID_50_) of ncp BVDV-2 or mock-infected mice. Data are presented as the mean ± SEM of three independent experiments. Statistical analyses were performed by one-way analysis of variance in GraphPad Prism 5.0 software; * *P* < 0.05, ** *P* < 0.01, and *** *P* < 0.001 as compared with the mock-infected group

Histopathological lesions were observed in Peyer’s patches, spleen, and bone marrow of ncp BVDV-2-infected mice in this experiment. No histological lesions were seen in mock-infected mice. Although viral antigen was detected in mesenteric lymph nodes, no pathological changes were observed in this tissue. Ncp BVDV-2-infected mice generally showed mild histopathological changes (Table 3). The histopathological lesions found in Peyer’s patches were a result of lympholysis (Fig. 4a). Mild lympholysis was consistently seen in high-dose-inoculated mice from day 2 pi through day 9 pi, whereas this lesion was not observed in low-dose-inoculated mice on day 2 pi but became apparent by day 9. In the spleen, lympholysis (Fig. 4c; blue arrow) and infiltration by inflammatory cells (mostly monocytes; Fig. 4c; black arrow) were evident in low- and high-dose-inoculated mice until days 9 pi. Histopathological changes in the spleen were greater in high-dose-inoculated mice than in low-dose-inoculated animals (Table 3). In bone marrow, the number of megakaryocytes increased relative to that in mock-infected mice. In low-dose-inoculated mice, megakaryocyte numbers increased significantly on day 5 pi (*P* < 0.01; Fig. 4e) and then decreased on day 9 pi (*P* < 0.05), whereas in high-dose-inoculated mice, the numbers of megakaryocytes on day 2 (*P* < 0.01) were higher than those in low-dose-inoculated mice, continually increased until day 5 (*P* < 0.001), and then slightly decreased on day 9 pi (*P* < 0.01; Fig. 5). The numbers of megakaryocytes were higher in high-dose-inoculated animals than in low-dose- and mock-infected mice during the experiment. The number of megakaryocytes was the highest on day 5 pi in both low- and high-dose-inoculated mice.

**Table 3:** A summary of results of histopathologic lesions in mice inoculated orally with a low or high dose of ncp BVDV-2 determined by IHC

Day	Day 2								Day 5								Day 9							
	L1	L2	L3	L4	H1	H2	H3	H4	L1	L2	L3	L4	H1	H2	H3	H4	L1	L2	L3	L4	H1	H2	H3	H4
Tissues																								
Lympholysis in Peyer’s patches	−	−	−	−	+	+	+	+	−	−	+	+	+	+	+	+	+	+	+	+	+	+	+	+
Lympholysis in the spleen	−	−	−	−	−	−	−	+	−	−	−	−	+	+	+	+	−	−	+	+	+	+	+	+
Inflammatory cells infiltration in the spleen	−	−	+	+	+	+	+	+	+	+	+	+	++	++	+	+	++	+	+	−	++	+	+	++

Eight mice were euthanized at each time point. Four mice were inoculated with a low dose, and four were inoculated with a high dose of the virus. Three independent experiments were conducted, and the results were the same. The data are representative of three independent experiments

L: low dose (4 × 10^5^ TCID_50_); H: high lose (1.2 × 10^6^ TCID_50_)

“−”: no lesion, “+”: mild lesions, “++”: moderate lesions

## Discussion

In this study, oral inoculation of mice with ncp BVDV-2 caused transient thrombocytopenia and leukopenia, the presence of viral RNA in the spleen by RT-PCR, and the presence of the viral antigen in the lymphocytes of BALT, bone marrow, mesenteric lymph nodes, Peyer’s patches, and spleen by IHC, despite the absence of clinical manifestations. Moreover, in ncp BVDV-2-inoculated mice, histopathological lesions such as lympholysis, infiltration by inflammatory cells, and a significant increase in megakaryocyte numbers were observed in Peyer’s patches, spleen, and bone marrow, respectively. These effects were more prominent in high-dose-inoculated mice. The present results show the establishment of ncp BVDV-2 infection in mice although thrombocytopenia, leukopenia, lymphopenia, detection of the viral antigen in various tissues, and the development of tissue lesions (as observed in cattle and ncp BVDV-1-infected mice) were less frequently seen in this study. Taken together, our findings provide insight into the pathogenesis of ncp BVDV-2 infection in mice.

We found that ncp BVDV-2-inoculated mice did not develop any clinical signs, such as ruffled fur, reduced activity, crouching, and reluctance to move, until the end of the experiment, and no weight loss was observed in these mice. This finding is different from what is seen in mice infected with ncp BVDV-1 [12]. Our recent study showed no clinical manifestations in mice orally inoculated with ncp BVDV-1 although ncp BVDV-1-inoculated mice lost weight [12]. It is difficult to explain why weight loss did not occur in mice infected with ncp BVDV-2 here. This phenomenon is probably associated with the limited ability of this virus to cause the infection.

Thrombocytopenia is a frequent effect of a viral infection, and viruses follow a variety of distinct strategies to decrease the numbers of circulating platelets [20–23]. Ncp BVDV-2 infection in mice here led to thrombocytopenia at the beginning of infection. This result is clearly different from that of ncp BVDV-1 infection, in which significant thrombocytopenia takes place [12]; on the other hand, our present findings are similar to characteristics of cp BVDV-1 infection [13]. In this study, the reason why thrombocytopenia was observed only at an early time point (day 2) is not clear; however, thrombocytopenia seems to be associated with virulence of the virus species. Virulent ncp BVDV-2 infection has been shown consistently to induce thrombocytopenia in cattle [24, 25]. Several studies have documented the outcomes of infections with different strains of BVDV in cattle, with clinical and necropsy results indicating different severity levels of infection, depending on virulence of the virus species [14, 15, 26–28]. Therefore, the transient thrombocytopenia observed in ncp BVDV-2-inoculated mice here suggests that the virus used in this study may be considered a low virulence species and that the consequent dissemination of this virus throughout the body may be restricted in ncp BVDV-2-infected mice.

The hematological hallmark of BVDV infection is leukopenia, primarily lymphopenia [29]. In the present study, lymphopenia was not observed in all ncp BVDV-2-infected mice throughout this experiment; however, a significant reduction in leukocytes was found in low-dose-inoculated animals on day 2. Our previous study of ncp BVDV-1-infected mice has revealed lymphopenia in low-lose-inoculated mice at early time points and in high- dose-inoculated animals on day 9 [12]. A decrease in leukocyte counts was not observed in all ncp BVDV-1-infected mice [12], but in ncp BVDV-2-infected animals, the numbers of circulating leukocytes were diminished. Unlike in ncp BVDV-1 infections, the numbers of other leukocytes did not increase in ncp BVDV-2-infected mice. We can speculate that the pathogenesis may be different between ncp BVDV-1 and ncp BVDV-2 infections in mice. Ridpath has reported that virulent BVDV-2 induces leukopenia and lymphopenia, whereas avirulent BVDV-2 decreases leukocyte numbers [24]. The reduction in leukocyte and lymphocyte counts was more frequently observed in low-dose- than in high-dose-inoculated mice in the present study although the difference in these counts was not statistically significant. These results suggest that the outcome of ncp BVDV-2 infection in mice is probably affected by the dose effect, resulting in the limited ability of ncp BVDV-2 to circulate in blood.

The viral antigen was consistently detected in lymphocytes of the spleen and bone marrow in all ncp BVDV-2-infected mice. Contrary to what was observed in ncp BVDV-1 infections, viral antigen was found in bone marrow and, less frequently, in mesenteric lymph nodes, Peyer’s patches, and BALT of ncp BVDV-2-infected mice. The viral antigens were found to be distributed evenly among these tissues, regardless of the virus dose (Table 2). In addition, the viral antigen was present for a limited time in mesenteric lymph nodes without induction of histopathological changes. Clearance of the virus from mesenteric lymph nodes, Peyer’s patches, and BALT was observed on day 5 pi. These data are quite different from those obtained in ncp BVDV-1 infections [12]. A previous study conducted by our group suggests that the viral antigen is present less frequently in the lymphoid tissues of mice injected intraperitoneally with ncp BVDV-2 when compared with ncp BVDV-1 [30], indicating that ncp BVDV-2 is much more restricted in its ability to spread and replicate in lymphoid tissues than ncp BVDV-1 is. Our findings suggest that the differences in antigen detection are related to the virulence of a virus species. Additionally, according to the results of RT-PCR, the spleen is the preferred target tissue for BVDV detection in ncp BVDV-2-infected mice.

A correlation between the distribution of the viral antigen and histopathological lesions was not apparent in this study. Although the viral antigen was less often detected in Peyer’s patches, lympholysis was observed there throughout the experiment. Unlike what was observed in ncp BVDV-1 infections, lymphoid depletion was not found in the mesenteric lymph nodes and Peyer’s patches of all ncp BVDV-2-infected mice. This result can be explained by rapid virus clearance, which prevented further spread throughout lymphoid tissues. Our findings suggest that ncp BVDV-2 infection does not cause lymphopenia in mice. As in ncp BVDV-1-infected mice, lymphoid depletion in the spleen was not observed in ncp BVDV-2-infected mice, but lympholysis in the spleen was observed. In addition, infiltration by inflammatory cells, especially monocytes, was apparent in the spleen. This result is in agreement with the characteristics of ncp BVDV-1 infections, and these findings were frequent in high-dose-inoculated mice. The cause of the increased infiltration by inflammatory cells in mice infected with ncp BVDV was not resolved in this study. This pathological change is likely specific to the murine model, not to cattle. Therefore, this phenomenon in the spleen may be a result of the host immune response against ncp BVDV infection.

In this study, the number of megakaryocytes increased significantly in bone marrow. Our previous studies have shown that cp BVDV-1 or ncp BVDV-1 infections in mice after oral inoculation result in a significant increase in the number of megakaryocytes in the spleen but not in bone marrow [12, 13]. The differences between these groups may be due to the virus species used. Consequently, our results indicate a significant increase in the number of megakaryocytes in BVDV-infected mice, irrespective of the virus species. The increase in megakaryocyte counts may be explained as compensation for an insufficient number of platelets under the influence of BVDV infection in mice. Despite the increases in megakaryocyte numbers, the viral antigen in the murine model was not detected by IHC in the megakaryocytes of bone marrow. In contrast to what was observed in mice here, a viral antigen in cattle was found in megakaryocytes [22, 31, 32]. This observation implies that the pathogenesis of BVDV infection may differ between cattle and mice. Thus, cells in bone marrow in which a viral antigen has been detected may serve as an indicator to distinguish BVDV-infected hosts (mice and cattle). Our results imply that thrombocytopenia in mice and cattle may ultimately be mediated by different mechanisms.

## Conclusions

Ncp BVDV-2 infection in mice after oral inoculation caused transient thrombocytopenia and leukopenia, limited viral antigen spread to lymphoid tissues, induced less severe histopathological lesions, and increased the number of megakaryocytes in bone marrow, in comparison with ncp BVDV-1-infected mice. In addition, ncp BVDV-2 infection in cattle has these distinctive features: lymphopenia, thrombocytopenia, lymphoid depletion in lymphatic organs, and distribution of the viral antigen among various tissues as well as the presence of the viral antigen in megakaryocytes, when compared to ncp BVDV-2-infected mice. These observations suggest that there are marked differences in pathogenesis according to host and virus species used for inoculation. In the present study, ncp BVDV-2 infection was established in mice despite the lack of hematological changes and histopathological lesions indicative of BVDV infection, which were relatively fewer than those in ncp BVDV-1. Overall, the ncp BVDV-1 infection in mice was more similar to cattle infection than to ncp BVDV-2 infection in mice in terms of the hematological and histological findings. We cannot be certain, but ncp BVDV-1 appears to be more suitable than ncp BVDV-2 for setting up a BVDV infection in a murine model. These data provide important information for understanding the differences in pathogenesis between BVDV-1 and BVDV-2 infections in mice. Further studies are therefore necessary to determine the biological significance of mice for BVDV infection.

## Abbreviations

BALT: Bronchus-associated lymphoid tissue
BVDV: Bovine viral diarrhea virus
cp: Cytopathic
IHC: Immunohistochemistry
MDBK: Madin-Darby bovine kidney
MLN: Mesenteric lymph node
ncp: Noncytopathic
PI: Persistent infection
PI: Postinfection
RT-PCR: Reverse transcription-polymerase chain reaction
TCID_50_: 50% tissue culture infective dose
